# Supplementary material for: What Was Old Is New Again: The Pennate Diatom Haslea ostrearia (Gaillon) Simonsen in the Multi-Omic Age
Source: Mar Drugs. 2022 Mar 29;20(4):234. doi: 10.3390/md20040234 (PMC9033121; doi:10.3390/md20040234)
Supplement: Supplementary file 1 [file marinedrugs-20-00234-s001.zip › marinedrugs-1608226-supplementary.pdf]

marine drugs

Supplementary Materials

Review

What was old is new again: the pennate diatom *Haslea ostrearia* (Gaillon) Simonsen at the multi-omic age

Noujoud Gabed [1,2,3], Frédéric Verret [1,*], Aurélie Peticca [4], Igor Kryvoruchko [5], Romain Gastineau [6], Orlane Bosson [4], Julie Séveno [4], Olga Davidovich [7] , Nikolai Davidovich [6,7], Andrzej Witkowski [6], Jon Bent [1], Amel Khitem [1], Efstathia Ioannou [8], Aikaterini Koutsaviti [8], Vassilios Roussis [8], Hélène Gâteau [4], Suliya Phimmaha [4], Vincent Leignel [4], Myriam Badawi [4], Feriel Khiar [4], Nellie Francezon [9], Mostefa Fodil [4], Pamela Pasetto [9] and Jean-Luc Mouget [4]

1 Institute of Marine Biology, Biotechnology and Aquaculture, Hellenic Centre for Marine Research (HCMR), 71003 Crete, Greece; gabednoujoud@yahoo.fr (N.G.); jonbent@hcmr.gr (J.B.); khitembenali24@gmail.com (A.K.)
2 Oran High School of Biological Sciences (ESSBO), Cellular and Molecular Biology Department, Oran 31000, Algeria; gabednoujoud@yahoo.fr (N.G.)
3 Laboratoire d'aquaculture et bioremediation AquaBior, université d'Oran1, Algeria; gabednoujoud@yahoo.fr (N.G.)
4 Laboratoire Biologie des Organismes, Santé, Environnement, Le Mans Université, Avenue Olivier Messiaen, 72085, Le Mans, France; Aurelie.Peticca@univ-lemans.fr (A.P.); orlane.bosson@univ-lemans.fr (O.B.); julie.seveno@univ-lemans.fr (J.S.); helene.gateau@univ-lemans.fr, (H.G.); Suliya.Phimmaha.Etu@univ-lemans.fr, (SP) ; vincent.leignel@univ-lemans.fr, (V.L.); Myriam.Badawi@univ-lemans.fr, (M.B.); feriel.Khiar@univ-lemans.fr, (F.K.); mostefa.fodil@univ-lemans.fr (M.F.); Jean-Luc.Mouget@univ-lemans.fr (J.-L.M.)
5 Biology Department, United Arab Emirates University (UAEU), Al Ain 33013, United Arab Emirates; igor.kryvoruchko@uaeu.ac.ae (I.K.)
6 Institute of Marine and Environmental Sciences, University of Szczecin, Mickiewicza 16, 70-383 Szczecin, Poland; romain.gastineau@usz.edu.pl (R.G.); nikolai.davidovich@usz.edu.pl (N.D.); andrzej.witkowski@usz.edu.pl (A.W.)
7 Karadag Scientific Station, Natural Reserve of the Russian Academy of Sciences, Kurortnoe, 98188 Feodosiya, Russia; olivdav@mail.ru, (O.D.); nickolaid@yandex.ru (N.D.)
8 Section of Pharmacognosy and Chemistry of Natural Products, Department of Pharmacy, National and Ka-podistrian University of Athens, Panepistimiopolis Zografou, Athens 15771, Greece; eioannou@pharm.uoa.gr (E.I.); kkoutsaviti@pharm.uoa.gr (Ai.K.); roussis@pharm.uoa.gr (V.R.).
9 Institut des Molécules et Matériaux du Mans, UMR CNRS 6283, Le Mans Université, Avenue Olivier Messiaen, 72085 Le Mans CEDEX 9, France; nellie.francezon.1@ulaval.ca (N.F.); pamela.pasetto@univ-lemans.fr (P.P.)
* Correspondence: fverret@hcmr.gr; Tel.: +30-2810-337-852

Citation: Gabed, N.; Verret, F.; Peticca, A. What was old is new again: the pennate diatom Haslea ostrearia (Gaillon) Simonsen at the multi-omic age. *Mar. Drugs* **2022**, *20*, x. https://doi.org/10.3390/xxxxx

Academic Editor(s): Leila Tirichine,Wim Vyverman,Detmer Sipkema

Received: 7 February 2022

Accepted: 18 March 2022

Published: 29 March 2022

Publisher's Note: MDPI stays neutral with regard to jurisdictional claims in published maps and institutional affiliations.

Copyright: © 2022 by the authors. Licensee MDPI, Basel, Switzerland. This article is an open access article distributed under the terms and conditions of the Creative Commons Attribution (CC BY) license (https://creativecommons.org/licenses/by/4.0/).

1. Supplementary Material and Methods S1: Experimental details on Frustules exploitation

The two required oligomers with a triethoxysilane group at a chain end and a hydroxyl group at the other (Mn 1900 g/mol, Đ 2.2) and the di-acrylate oligoisoprenes (Mn 4100 g/mol, Đ 2.1) were synthesized from solid natural rubber following a procedure described in Tran *et al.* 2021. Model mesoporous silica particles were synthesized dissolving 12 g of hexadecyl trimethyl ammonium bromide (1 eq.) and 16,1 g of tetraethylorthosilicate (1,3 eq.) in 250 mL of ethanol and adding a solution of 65 mL of NH_4OH in 200 mL H_2O. The solution was stirred for 1h at RT, then the whitish suspension was filtered and the silica particles were washed with 150 mL of ethanol, dried at 80°C overnight and calcined in an oven at 500 ^{o}C during 24h. TEM images showed that the particles had a perfect spherical morphology, with 150 nm average diameter and pores oriented perpendicularly to the surface, with 21 Å pore diameter (Figure 12 main text). The elimination of the organic matter from *Haslea ostrearia* was carried out by treating the dry biomass (2 g) at RT overnight with a mixture of concentrated nitric and sulfuric acid (30 mL of each), then washing with distilled water until pH 7. The frustules were dried at 80°C and TEM micrographs showed debris with a dispersion of sizes and morphology, with complete elimination of the organic content even from inside the squares of the regular network. BET measures showed that the silica of the frustules is mesoporous too, with 0.35 cm^3/g pore volume and 41 Å pore diameter. The grafting conditions were optimized using the mesoporous silica particles (0.5 g) and incubating them in dry toluene (10 mL), at reflux overnight, under argon, in suspension in a 0.09 M solution of silane-oligomers (1.5 g in 10 mL toluene). After filtration, the functionalized particles were extensively washed with dichloromethane and dried in an oven at 80°C. The same procedure was used with the frustules debris. BET measurements showed that the specific surface for the mesoporous particles diminished from 1269 m^2/g to 425 m^2/g after grafting, while for the frustules decreased from 336 m^2/g to 6.4 m^2/g. TEM images confirmed that both synthetic and biosilica were surrounded by a layer of polymer coating (Figure 12 main text). Thick films were obtained by mixing 200 mg of di-acrylate oligomers and 1 or 5% weight of silica particles, in presence of 5% weight of Darocure photinitiator, and irradiating the formulation deposited in teflon molds (1 cm diameter, 0.5 cm depth) for 10 minutes under a UV-lamp (intensity ~29,4 mW/cm^2). Visual observation showed that both bare synthetic and biosilica particles formed aggregates in the film volume, while functionalized particles were more easily dispersed, confirming the initial hypothesis that the rubber thin layer would improve dispersion in the rubber matrix. Of course, the silica frustules will not replace carbon black or silica as charges in elastomers applications such as joints or tyres but this study showed that the biosilica can give the same chemistry as the synthetic one, opening the way for other interesting applications.

2. Supplementary Information S1: Sequence of *H. ostrearia* DNMT homologues

```
>TRINITY_GG_4998_c0_g1_i2 len=2651 path=[0:0-858 1:859-933 2:934-1694 4:1695-2650] Gene_family="DNMT3"
TTGACATCAATATTCTTGTGGAAATAGTATAGTAAATAGCTAAGTCTCTTGTTTTCAAAACACGAAGCA
TCAATCTATCCACGTGCCCGGAATTATCTCCACCGATGATTTCTTCGACCTGAACCAAGCAACATAAT
ACATGAAGCCATCAGAAAGAAAGTAGGCCGTCGTAATCACCATGACGAAATTCAAATAAGTCTATAT
GTATGTCTAATAGAGGAAACAAACATAATCCGATTTCAAAACAAAGTCAATCAATGACTTTTAGTGCC
TTCAAATCCGAGATATCCTATGCTTCCCTTTCTTGTTCCATGTGCCGATTCCACTCAACACAAATAGAT
AAATCTTCATATAAATTCAGCCATACCCGGCATACCTGCCCTACCACGAGAAACATATCGAGCACTAT
GTTCCTCAGTATCGTATTCAGATTCACTCGTTGCGGAATCATCGATAGAATCTTCCCAGGCAAAGTTGT
AATCATATCCTTTGTATTCTCTTGAGGCAAAGTATCTTTGAAGTGGTCTCAATATGATGGCGACTGTTG
GAATTGAAAAGGAATTACCAATCAACCGCTTAGAATATTGGTCGACATTGTAGTATGATGGAGAAGTC
GTCGTCATTGGTGGTGTCATCTTCATACTTGGGGTAGTCCATTTAAACGCGTCAAATTTGTACGACTGA
CAACGATCTGGTAGTCTATGGTAGTTGCCAGCAAAGGTATGGTACTTTTTTGGGAGTTTTTCCTTCCAG
GCTAGTTCAGTGTCAAATAGAACCCCAAGCGCGTTTGTCAAAAGACTGCCAAATAGCTGATCCATTGG
CTCTTTCACGTAATTTAGGGGCATACCCATCATTCGCTTTGAGTTGAAAGAGGCCAGAAAGTGAGACT
```

TCAATTGCAAACTCTACGTTCCCATACGAATTGATAAACCCACCTCCCTTTCTTGTACAGTTATATGCC
TTCCTACAAATTGTTTGTCGCCTTTCTTAAACACCAACATCCTTTCATCATTTACCCTGCCCAACGACGC
CATAAAGCACTGCATTTTCGCAACCATGTCAGGCTCGAAGAAATGTTTAGCAAGTTGAAAGTCGTCAT
CAAGGCAAGTGCTTGCAGCAGGATCCTTCATGTCCTCTGCCTTGATACCCACTGGAATGTTGGTGAGA
AACGTTCGTTTCCGACGGCATGGTGAATAGTCGCTTGCATCTAGAGTAATTGACCACTCTATATCGTAT
GCTTGCTCGATTGTGTGGAGTTCGCCCGATTCAATAGGTTCGTTTGCACTGTTGGAAAGACGCGTGTTC
TCTCCCATGAAAAATAATGGTTTGGATTGTTTTCTCTTAATCTGTTGTAACAACTGCCCGAAGCGAACC
ATGTAGCTCCCTTGGTTTCCACGTGTACCTTGGCGGCTGGCATTCACTCCTGAATAGTCGACGCACGGT
GGACCCCCCACGACAAGGTCAATCGGTCCATGCTTTAGTAAAAAAGTCTCCAGAAATTCTTCTAAATG
TTCAAAGGTCGTATAGTAAACATGTTCAATGCCATCATCGTCCAGTTCAGTGTTGTATGAGCAATCATG
GTTATGCTTATAAACATGAGTTGCAACCTTATCATGCTCCACATGAATTACTTTCTTCATGGCTATTCCA
AGGCGTTTAAGACAAATGATCGCAGATCCAATTCCAGCAAAACAATCCAAGACAACAGCAGCTCCCT
TTTTTTCTAGGTTGCAATGTTTTTCGAAAAGTTTCATATTCGTGCAGTAGGATTGATTCGTTTGAAGTAC
TGAACACGGACTAGGTAGGCTGTCTGCATTGTACATTTCGGATTCTTGACTTTTCAAGGTTCGATACTTT
TGGAGAACATCTGCCCTGGAAAACGTTTCCTCGTCTGATATTCGACTATTTTCAAAAAACAATGGTTCA
GAGGAGTCATATGCACTATTCATAGCTTCCTCGACAGGAGCTAGCCATTCTTCAGAAGGATTAGTCAC
ATTATGGCAACATCTTCGATGCCAGAGAGAGTTATCACCAGAGTGAACAAAACTGTCCTTTCTAAATT
TCTTGAAGTGTGCTGGCGAACATTCACAGCGGTAACAACAAAAGGCCAGTTTGTCTTCAATTTCCTCA
ACCTCGTAGTGCACACCGGGTAACGTCCCCACTTCCATTCGACAATTCCAAGCATGCTTTCGTGAAGCT
TTCTGTCGTTTTGCTGCCGGCTCATAAGAAACATCGCCATAATCATCTTCTGAATCAGAAGGGTCCGCG
TTTCGCACAGCAACATTCTTCTGTGGCCTTGTTCTCATGATCCCCCTCTCTTTCTTCAAGTTACTATTATT
CTTCTGTCGAACTCTTCTACTCGATCTATCACTCTTTCTCCAAAAACTGTGCCGATTGAACTTTATTAGG
CCAGCCTTCCTAACGAGTGCTAAGATGCTTCACACGTTGCAGATGGATAATCCCACCTTCGATCCGAC
TTTCTTCTCAAGGTGATGAGATGTGATTTGAAGAATGTCGAAAGTATAGTAAAGTGCGATCGGACAAT
GTTGTGTTTCTACCTTGGATGAAATTGAGTAGAGTATAATAAATTACTACATACACGTGGTCTTGTGGT
GGCAGTAGAAGTCGCAAAAGAAAGAAAAGAAGAAACGCTC
>TRINITY_GG_3357_c18_g1_i2 len=6743 path=[0:0-5174 1:5175-5245 2:5246-6742] Gene_family="DNMT5"
TTTTTTTTCATCATAAACAACGACTATTAATATTTAGCGTATCACGTAACATAGATTGACGATCCTGAA
TCACTGTAACTCCCATAGGCACTGCTTGGCCATAATGCAACCCCACCCCCTCTCTCAGATGCTTTGTTT
AATTATTCGTCACCAGCGTTGGTTGCTTTGGAAATGATTTCCTCGTCCGTATTCCTTCGTAACAGTTGCC
CGTCAAGACATTCTTCTCCGTTCGGCCCTTCAACCTCAATTCGATATACGTTCACGAGAGGTCTTAGTT
GACCAGCACGAAATACTCTACCAATCGCCTGCTGCTCGGTACTGGCATCAGCCACAGGATCTGAACTT
GAACCACCTGAACCCACGTATAACGGACTGAATAGGACCAAGTTGTAGCACTCGGTTTGTAAGTTCAA
ACCTGCTGCGTGCTCAAAGTGCAAGATTAGTATGCGAGGACGTCTTCGATCTTCTTCTGTGGCATCACC
TTGTTGATACCAGGAGATTTTCTTATTCTTCATGGCAACCGAATCAGTTTGCTCGAGCCATGTGCAACC
AAGACCGCTCTCTTCCAAGGAATCAATAGCAGCTTGCCCAGCCCCAATACGACCATCTGTAAAGACC
ACTATTTTTGTTTTTGGGTCATCTTTTAGGACTTTCTGACAAATTTCCACCAATTTCTTGCCTCGGCTCTC
CAAACTTGAATTCTGTCTCAGGAACCGGGTGATATCATGACCAGCAGACACATCAAAATCCTCGGGTT
TGATTGCAAGACACCGTTTTGCTTGACTAATTCCACTTTGACTCTTAAGGTACAAAGCTTCCTCTTCTTC
GGACATTTTCACCGTACAGCGGATTTCACGCTTCTTGATTCCATTCATTTCCGCTCCTGCCTTGTTGCGG
CAACAAGCAACTTTCAAGTACTCCTGGCATTTTGAAAAGATGGCTTTGCGAATCTCGCGACCTTGCTTT
GGTTCCAGATAGTTCTGCAAGAAAATGTCGCGGCATGATTCTCGCTCTAACTTTCGCCAATGAGAAGA
CAGTCCGATTACATAGACGCCTCCCATAAGATTTGCCAACTCAATAACTCTGTCACTGCTGTCAAGCA
ATGGCGTTCCGGTTAAACCAAACACAGCTCTGCGGCATACAAGAGGTCGAAGACTTGGGTTTCTTTGT
GTTATGCCAAGGAACTCACGCCCTGCTCTCCGATTCTTTTCCTTGAAGAAACCTGAGTCGCTGTCCTTT
GTTGCTTCCTTTGCCTCTTTTATTTCAGATTTTGTTGTGCACAAGCATTCATGAATCTCATCAACGAATAT
TCGCTCAAATTCAAAATATTCCAGCGGAACACCTTTCTCATAGGATTGAAACTGACGCTTCCTTAACGC
CACAATAGCTTTTTGGTACGTGTACGTATAGAAGGCAGATTGATCCCTCCTTCGCTGTCCTCTTGGATT
GTTTCCTCCGCCATATGGATCTGTTGATGTAGCAGGGATCCACAGTCCTCGCGCAGCTCGTTGCTCCAG
TTGACCATCGTAATGTGGAAGAACGGGCAAATGTTCGGTGTCGATCTCAACTTTTGACTTCTGCACAA
GATTCTTGAAATATCCAGCTGATTCGAGAATATCAACGGGACAAATAATTATGTCAGCTTTCATAATG
GTTTCAGCAGATGTGACCTTTAGAGCTGCGAAATCATAAATTTTCAACACAGTCAAGCCGCTAGTAAA
CTTTCCAACTTCACTTTCCCATTGATCGATAAGTGCAGGCGGGACAACCACAATCGTCGCCCCGCTTTT
CCTTGGCTTGGCGCGTGCTGCTCTAGCACGAGCAATCCCTTGTAGAATTATGGCAATTGAGATTACCGT
CTTTCCGGCACCAATCGCGTCAGCAATTACACCACCTTTGACTTGGGTTGGCATAGTAGCCTTGGCAAT
TAACGATATTCCCGCAGCACCAGGCATATCAAACTCGCTCATTTCAATCTCGTCGTATTTTCTCTGTTCG
TCCTCAATCGACAACATTTTTGTCACAACTTTTTGTTGTCGTTCATACAGACGGAAAGGTCCTTCGAGA
TTAACTTCCGTGTTTCTTTCACCACTGCAATTGCTGACAACGAATGGAGTGACAATGGGATCGCTTTGC
TGAGTAATATCAGAGAGCTTAAAGCTTACCGTTAGATTCTGTCCAAAGGAATCCCCTCCACGACCTCT
AATGAGGTGTCCCGCTGCATGATGTGCTGCGACTGAAGGGTCGCAGCTGATGGTAATGCTCCGAGCTT
CAGTGTCAAGCTCAAATTCGAAGGTGCGAGGTGCTTCTTGCAGCTGCGAGTAGTACTTGCGTGCTGCT
CCAGGGTCACTGTTTCTCTCGTAGAGTCCTGCTTGTTTGCGTTCATGGTCATAAGCATCCTCGTTGAACT
CCCACTTCTCCGTTGGACGGATTGGGACAGTCTTGCAACAACGCCCAAATGAAACAGAGTCAGTTGGT
TCCACTACTTCCCAACCTGCTGCTGGAGACAAATCATACTTGAAGCCACCAGATGCTGCTAGCTGTAG
CAATGGAGCAACACAAACAGAATTGAGGACACGAAGCACATGCTGTACATGATTCCCTCCTTTCAGA
TTCAATTTCATGCGGTTCGTTTGTTGCATCCCCTGACTTAGAAGTGTTACATTATCTTTCGAAAGCCCAG
AAATCTTTGCAAGTACGTTTGATTGATCACTGCCCTTTGACACTGATAATGATGACTCCGGGATTAGGC
ATTGCATCTTCGGCAGGATTGTCCATTCCCTAAAGGCTAGACTCAACTTGCCAGATTCGAAAGAATCA
CAGGGTTCCCAGAATTCCGGGAACTCGGCTATGATGGAAGATAGGTCGTCGTGGTCACCAGACAGGG

AAATTATTGCTCTATCTGGACCAGTGCGGCTAACGTTAGGCCGCATAAGTAAGTACAATGATGGACGT
CCGCCTGAAGCCTTTTTAATGTAAAGCGCAGATTGGTTGGTGGTCTGCCGACAAGCTGCACGGTCATA
ACTTCCGAAGAGATCAAGCAAATCCGAGCCTGCTGGTTTAATCTCGATTCTCTGTGGCCACTTCTTCCA
TCCATTAGAATACACCCATCTTCGTTGCTCACCTCTTTCCTTCGCATTCGGAAAGTCCTTTTTGTTCGTTT
TCAGCGCTTTCTTGATGTCATCCAACACTGTTTCTTTGAGCCCAACCTCAATGCGTGGTGATGGAGTTGT
TTCCTCGCCACTGACAACAACATCAATCTCTCTAGATAATCCCATATTGCCCTGCCATTCTTTAGAAGA
GTCATGAAGATCATTCTTCATCGAAATAATAAGCTTTGCACAGGGTTCGAGCTTACCAAACACCAACG
GTTCTGTTCTAGCAGGAACAAAAGAGGTGATTTCTGCCATCAAACCCACTGATATCGACTCTTGATTCA
CAACTTCTTCGCGTTTCAATTCACCGATGGTAAGGCGGAGTTCAGCCTGTGTCACACCAACGTTTTTGT
TGTCTCGTGCATAATATACGACAACCCATTTTCTCCGTTCTCTTTTCACACGATGCAAATTGAAGGATC
TATGCTTAAGTGATGCGACCCTATGCAAATCTTCACGAAGAGCGCGGATATCATCCCATCCTTCTGAA
GATACCATTAACGTGTGCGGTAAGACTTCTCGAAGCTTTGTTTGGAACGTTCCAAGGTTGTGCTCAGGT
TCAGAAATATCAACCGAACACGTGTCATGTGATGACATGTTGTAGCCAGATACTTGGTTGACACAATT
TCTGCAACACGAGACGCGACATAATCGACACTGTAGAAATGCAGAAGTGCGGGAATTGCTACCACTG
GTCTCGCAAGTGCACCATATGGAGGTTTTCACTGCATCCGGAGCCAAGGAAGCTAGTCGTTTGAACAA
CTCTAGAGCGTTCGACATTGACGCCTCCGTTGGTACGTCATCGGGACAAACTAACGACAAATCTGTCT
TCGAGCTCGTTCTGTGTTCTTCTTTCAGCAGCTTTTTCGCCTCTTGCAGAGTACCACACTCCTGACGTAG
CTGTTGACACGTAATTGCCGCCAAGATAGTCGATGAGACTACAGTGAGGCTCATCGCATTGCCCGCAA
GATCTCCAAGCTGTACTTCAGTCTCTGTACCAAGAATCAGTCTTTCATATGGGATTCCCTGCATCAAGA
GCTTTTCACAGCCCAACAACGGGCGCCCCTTGTGTGGAACTACAAAGTCACCACCTGGAGTTACACAG
CCGGCAACACCGGGAGTTGCGGCCCTATGATGTTCTTTAGAGGCATTTTGCGAAATATTCCAGAAGTA
CGAGCAGAAATTTGGGTCGTGAACTTCTGCATCCCTAAAGGCCGAAGCATACAATATGTCTAACATGT
CGATTTCTCTTTGAAAGTTGCAGTCTAGATATTCGATCCAGAAATGAGGAGGAACTTTCTTTTGTCCGT
AAGGCCCCCATTGAGTGAACCAACGGGCTCTATCTTCCAAACCAAGGTTTTTTCGCGTGTGCTTGTTAT
GAGGCAGATTGGCGCTTGCCGAAGTCCAAAAGTCAGACTCAAGGAAGCACTTTCTTTTGGAGATTCGG
CCTGCAGGTCCATTGAGCGCCTCTCGAAATTCTCGAATTTTTTCGTGATCAACTTCCAACATAAAGGCG
TCCATTGAATGTCTCGCGGGTGTTCGTAGATGTTCAATGATTTCTCTCCAGTAGGCACCAAGATCGTCG
TAGACATTTCCATCCTGTGGTTGCCATACAAACATGTAGACTCGTTGCCGAGTTTGAGGAAGCCCAAA
GTCCTTGGTATCAACTGATATAGTTTGAGTGCAATAGGTACATTCTGTCTCAAAGACTAGGGTATCGCT
GGATTTTGATATACCGTTTTCTTTTATGAGTTCAGTAAGCGTACACTTAGTTGCGCATTTCGATGGCCAC
CGAACGGGATTTAGCGTCAAAGAATGTGGCTGAGTATATCCTTGAAGAACTGCACCACAACGTACGC
CATAAACGGCAGGAACCGACTCAACAATAATTGCCTTGTCGGCCCTGCTGTATGAGAATTCGAGGTCT
TTTTTGGCATCCTTCCCTATGTTCTTGATCGCCTTAGTACTTGAACTTTCAGAGAGCTTGACTCGTCCTGT
GATATATTCCCTCATCTTGTCCTACAGCAACCAAATCACACGTGTTAAAACAAATGGAAGTACCTCAC
CAACGTGCTCACATTTAAACTTACCCAAGGAGCTAGTTTGACGTTCTCAAAAATCGCGAATGGTGGCC
TTTCTTTGAATAGAATTTCAACTGCAGCTAGAAATGTTTCTCCTGAGCAACCCTTATCCTCAATGTCAAT
ACGCCATTTTGAACGCATCATCGAAAAGTTTTTGCAGGATGTTCCGGCAATAAAGAGATTGAAGTCTG
GGATTGGCTTCTCTTGACCATAGACATCCCTAGGGACGTAAGTCGCAACATCCTTTCCATTCTCATCTTT
GCATGTGAGCTTAGTGATATCAGGATACAAAACTGAATCGAAGTTTCGTGCCAAATAGGCCTGTTTGA
AGGGGTCATTTTCACAAGAGAACTCGTGTGTATGGACAAAATCAGCGACGCTTCTTAGTTGCAATTGC
TCTTGAATCATAGTCAAAGCAAGGGCGGGTGCATCAGTTCCACTACAGGCAGTTCCAAGCTTCAGAGC
GTGGTTTTTGGTCCCTAGAGGATCTCCTTTGGCGTGTAGGGTTCTCTTGAGCAACGGAAGAAGCTTATC
CACCTGCTGGGAAACGAGATCATTAATAATATCATTCCCACCATCAATACCACCTTGATCCAGCGCAC
TCTTGTTCTTCTGAATCTCCTCCAAAGAAAACTTGGCATAGGATGGCGCAATCATCGGTTTGAACATCT
CGGCCATCTTGGGCTTACCAGTGCTTGCCTTTGCCGCAGGCTTTGTTGTCACTTTTGTAGTTGTCTTTTTT
GGCTTGGCCCGCTTTGGTTCTTCGGGCTCCGCTTCCTCTTCGTCCGATTCTGTCAACATTGGGAGGTCGT
GATCATCGGCGCGGTCGCCTTCAACCATAGAGAAGTCGGAGTCATCATCAGCATCACCGCCAATATCT
GAATCGGAATCAAGTAAGACTACCCTCTTCTTCTTTGAACCTCTTGTCGAGGTCAAAGACAGGCTCCT
ACTTTCTCGGACCGTGCGTTTCCGTTTCTGAGTTTGGACTGCCTTCTTTCTCAAGGTGAATGCTTCACCA
TTTTGCTTGTAGGAATTACTCCTGACGTCATCGACTGCATCCAAAGGCTCGTCCGAATCACTTTCCCGA
GATACAGCTCGCAGCCCCTCGTCCTCAATGTTTGGATCAGGTTCCCGTTTCATTCCCTCCTCAAGCTTA
GTGGACTTACTTGCTTTACGTTTGCCTCGCCGCGACTTTTTCAAAAACATGGATATCTCGTCCTGCTCCA
AGTCTTCTTCGTCGCCATCATCGTATCTAACTCTGTAGACCCACTCGCCTCCATCCAGCTTGTCGTAGG
AAATTATTTTCCCTGAGAACGGTTCCGGCTTCCCCGTCTTTGGATCAAAAAACCCACGCTCTATCTCGG
TACCCACATCGTACTTCTGGGAGTGGGATCCGTCGTTCATCGTTGCTTGTTTCCTCTCTAGTCGTTTCCG
AGTTTGCCCAAAAAATCTTTAACCTGGGGCACTCTGAGGAGTAGTCAATTGCTGCCAATGCGCTTTCC
GGAGA
>TRINITY_GG_3317_c13_g1_i2 len=4655 path=[0:0-3730 2:3731-4654] Gene_family="Bacterial-like DNMT"
CATCTGCTCGATCTTGGATGTCGCTAGCGAATTTTCGGCGGTCAAAGACAAGTTCACAACTTTCACAGC
CCACAGAAGTAGTAGGGTTCACGTTTCTAGACTCTCTCTCTATCTTTAAAAGATAGAGCGAATAGGAA
AGGATATAACAGAAAACTGGATCTAAAAGATTAAAAAAGAGAAAGCTATTCGGAAGTCTTCCCCTCC
TAATAGTGCAGTACGCCTCCTTCCAAGCCGTGGCTTCGAATGTTGTCAAATACAGTGCAAAGAATGTT
CTCGCTTGTGCCTGCCTTTTAAAAAAATCTGTTTGGAAGTAGTGAAGCAGTACAGAGCTTCTTCAGAGC
TATTGGTTCCTGCTTGACGCAGACCAGCCTGATTCATTCGAGCACTTTTCTATCTCTGTCTCACCAATTT
CCTCGTCTATGGATCCCATTTGCTCCACTCTTGGTCGAAACGCTCGATAAAGTCTTCAAGCGATTCGTC
GTCATCGAGGTCATCGTCGTCTTCCACTGCTTCTTCCAACAGCCGTTCTATTCTCTTGTGGTACTCTCCTT
TGGTATTGGGGGCGCCAGCATCAGCTGGAGACCAAACATAACTATCATCAAACCAATCGCCCCATTC
AGCTTCCAACCTAGCAAACGTTTCTTGCACGCTTTCTTCGCCTTCCTGAACCAGTTCAGGAACCTCGGG
CACAGCCGCTGTTTCGGTCTCTTCGCTCTTTGGAACTGTTTCTTCGATTGTCGTAGTCATTGCCGTCATGT

CCGTGCCTTTCTCTTCCCGTTCCTCCGCAAACTTATCTATATTTCTGCAATGGTTTCTGCAGAGACAG
CGAGATTCTTCCTTCTACATTAACGTCGCGACTCGACAGTCGAAGTAGATTGGTCGTGATTCGTTTGGT
TGATTCGGCAATTTTATGTTCCATTACCGCTGCTTCGTGCAGGTCCTGTTGAAGAGTCTGACAAACCTT
GTATAACTCCGTATTGCTCATGGATGAAATCGAAAAGGTATCTTCGGTTGACTTATCATCGCCCTGCGT
GTTCTCGTTACCTTTGGATCGTGCTTCCATTTCATCAACCACAGCCAGCAAACGGCCACGAATGATTGC
CTTTCGCTTAGAGGATGAAGCCAAGAGCAACGTCATGAACTGCTCTTGTGCAGAATATTCAGCCTTTCT
TTTCATTTCTTTCGAAATGATGATTCTCCCTCATCGGAATCAGTTGTAGCACTTGTGTCTGCAACAAAA
AAAGAATCCAGCAAACCCACGCAATCAAACATGTGCACAGAATTGGCTGCAACACCTTGGCCAACCG
CCGACGATGATGGTCCCTCAGAAGCAGCTTTCTTTACATCTACAAGATATGAAGAAGTTGTCTCATCAT
TATTCGCCTTGTCTAAAGGTTTCAAATCATCAGAAACGCCTAAAACATGTTCGTGTGATGACGACACA
TCACAAAAGGCATCATCAGGAACATCCTCACGCTTTGCCAACAGCAATCGCAAGTCTTCAGGACTAGT
TGCCGGGATGCCGTCGGATTCTCGTACAAGACATGAAAATAACCACTGCTTTGCAAGATTAGTCCATA
GAATTTCAATGCTTTGACTTCCGATGGAATAAATGGCATCCAGGGAACTCTTAACCGCATTTCGTGCCG
CAGGATGCTGTACCAATGTTCGAAGCAAGGAATCCTCAGCCCGTGCTACCCGTGAATCAACGTCTTTT
GCCAAAACCATGGCAGCCACACCATATTGAAGGATGGATGACTCTCTTTTTTCTTCTCTTGAATCAGAA
TAGCCAGCTAAACGACCCGATTCCGTCAAAAATTGATGACCATGATAGTCGTTCGAATTAAAATAGAC
GAGCTTTTCCTTTCCCTTACGTTGCGCTTGTGAAGCTCCAAGGGTGAGTGCTCCTCTATCTCTTTTCCAC
TTTTGTTTGGGAATTCCTAACCAATCAGCATCGGTTTGGTGCTTGAATTGTAGACCTGAAAAGTGTGGT
GTAATGGGAGGCTCCGGTTCTTTCAATTGGGCATTCTTTCGAAACAATTTCTTCCGAGCATCTTTTCCCT
TTGGACGGGAGACGTACTGGCCTTCGTCCTTTAGTGAAGACTTTGTCTTGTTGGCGCGTGCTTTCCAAG
ATAACTTGTCAATGTCGTCATTTGCTTCAAGTCCTTGATTCAATGCATTCAGAGTCATTTGAACTCCGTA
CGGAGAACTTGTTTCGTTACGTTCTTCTTGTTTCTGTTCGATGCGCCGTTGCATGGCTAGTTTCTCCTTTG
CAATACGAGCTGATAGATCAATCAGATCAGACTCCAAATGTAGACAAACGGCTTCCGAGCGAGCAGC
AGCGTCATTGCTCGCGCTACTGCTGCTATTACTGGAAGCCAAGAGATCTGCAAGAGAACCCGCCATAT
TGTAGGCCGCTTTGGTTCGTTCGGCAGGATTTGGGAGTGTCATGGCAAGAAACTGTGCGACTCTTGTAA
ATATATTCCCAAAACTGCCAAGGCTCTCCCGTGGAGCCTCGGGGTCATACTGTACAAGGATGGATTGT
AAAAACCAATCCGTCCAATCCAACGAATTATCCAGTACGGTGGTGCGAAACGCTGTTTCATCATCCAC
GTCACTCTGCTTTTTCTTGCTCTTCATTTTGCTTGTTTCAAGCGTATGTTCTCTTGGTCGTTGTTGTTGTCG
TTCTCTTTGTCGCTGTTCAATAAAGTCTGCTGGATCATGACAATGATCTGGAAGGCTGGCGACAAAGA
ACTCTACGCCGCATTGTGCTGCTACCTTGGATAGCATTCCACCCGAACAAATCCGCTCGACAGCTGCT
GTCCCAGCGGAATCATTGTCCATACATAGAATGACACGACCATTATGACGCGATCGTGCCAATTTGGC
AGCCGCTGTCAGTTGTTGTGTTGTAATGGCCGTTCCCATGGTAGCTACAACTTGCTGAATGCCTGCTTC
ATGCAATGTCATGGCATCCATATATCCTTCAACCATGACGAGTGGATTCTTCGTTTCCTCGTTTTTTCTC
GGTGCCTTGCGCGCCGTTTCCATCATTCTCGCACAAGACAATCCAAACAAAACATTCTTTTTTTGAAAA
ATGAGTGTTTCTGGACTATTTAGATATTTGGGTGGCTTATAGACGGTCGTACTATTGTCGGTTGTTGCTG
TCGCCTGTGAAAAGAGTGGAATTAGGCGTCCACCAAATCCAAGAACCCGATCGCCCGTCTCGTCCCA
AATGGGTACCATCATTCTGGCGCGAAACCGATCCATCAAGGAAGAATAATCCAAAAGGGGTTGTTGG
ACAATCGTTGTATTCTTTGTGCTGCTGGATGTACCACTTTTATGACTTGTTCGAGTTGTTGTCACAGAAT
TAGCATTGGATTGTTTTCGAATAGCCAAACCAGAGTCGAGAATTTCTTGGGGCGTAAAACCCTGTTGCT
GCAAATACTGAACCAATGTTGGTTGGCGTTGTCCTCTCTGTTGTCGATAGTGTGAATCAAAGTATACAT
CAGGTGCATATCCCATCAAAAATGTTCGAACAGTTGATGGAGACAATCCTCTGGATTGAAGATAATGC
CGTGCTGGACCACCCATCAAGTCAGTCAGGCACGTGTTATAATAGGATGCTGCTACGGAGTTTGCCAA
ATAAAGACGTTCTTTTTCTTCTTGTTGTTTTAAGTTGATCTTGACGCCGCGCGGAACAGACTTGGTCATG
GACATTATCTCATCGTAGACGGGAAACGGTGATCCTGGAACAAATCGTTGGAGTGCCATGATTGCTTG
TGAGAAAGACAAGGGCTGATTATGCACTGTTTGAGAATAACTTCGAACAAACTGAAAGACATCTCCA
CCTGCATCACAGACAAAGCACTTGTACATTCGGCGCTCTCCATCAATGGATAGGGATGGATTATGATC
ATCATGAAAGGGGCAAATAGCCTTGGCTCGTTTGCCATCAGGGAGGCGTTGAAATTGAGGCAATCCA
AAGGATTCCACCACATCTACAATGTCTGTCGAGTCTTTAATATCCTGCCAGGAGGAAGACTGCTGCTG
ATGTAGTGCTGGTACTAGTAGCCGTCGTTGTTGGTTGGCATTTCTATCATAGCGGTGCTGTTGTGAATGT
TTTGGTATGGAATACTGTGGGGAGAGAACTGGAAAACTAGGAGAAATCCAGGCAGTTGCTGGCTCCT
GCATGTTCAGTAGAATAGATATGCATAATAGCCACACCAACCATGCCTCCTTGCCTCTACTGCTTCTTC
TCTGTTTGCTGCTGTTAGGGGTAGTCACAGCAGTGAATACAACATCCTCGTCAACATTCTTTGTAGGCC
TCCAGTGCCACTCTACTCTTCGTCTCAATGTCGCTTGCAACGATTTCATCATTTGTTTGCGTCTATAGGA
TGTGCACAATAGCAAGAGATGTGTGCAATCGATGGATCGATGTATTGGAACAGAACTGTAAGCCAAG
TTTCCGCCTTATTGGAGCTTCCGATAATGATGGGGCGGGAGATCCTTGTTCAAGCTATATAAGAGATAC
GAATGGCACAACAACACCCCAGACACCTCTTGTCAGAGGGTGGCGATAGTGATTGCTTCTATCGCGAT
TGTGTTTCCGTGGAGTAAGTGTTCCCCACATACAAACCACAATGCTTGCT
>TRINITY_GG_1357_c0_g1_i1 len=1771 path=[0:0-108 1:109-120 2:121-1770] Gene_family="Bacterial-like DNMT"
CGCAACCTTGTTGGCACACGACTGTGTGGGACGCAAGTGGCATCTCGAGGGACGCTACGGCTCGCAA
ACCTTTGTCGTAGACGATGTCCACATGGGCCTGCCCTTGTTGTCCAATGCGGTGGCGGCAGTGGTAGCT
CGAAACATGACAACAGACAGGCAGTTCGAATCCGAACAACTGCAAGACTTGCTGGCCACGGATCAA
GTGGAAATTAGGTACCTGCCAGTTCCTATTCCTCGATTTGTCGACAAGCTGGAAACAATCGTCGACGC
GACCGCCCACGCCGATCGTAAACCTGTGCCTCTTCCCATGAAACTTCCAATGAGAACGCTACCGAACA
GAATCAGAAGCGACCCTCCGGCCTTTACGTTTGTAGACCTCTTTTGTGGAATTGGAGGGTTCGCTGTCG
CACTGGAAGCGCTGGGAGGTCAGTGCGCCTTTGCCTCGGACATTGACGAATCTTGTCGGGAAATGTAC
GTGGAAAACACAAGTGTCTCTCCTGGAGACGTCTACGGGGACATTTATCAAGTACCCGATTCAGAGTT
GCCAAGCAGGAATAATGTTGATCTCTTGGTGGGTGGCTTTCCCTGCCAGCCCTTTTCCTCGCTCGGCGA
GCAACCGGGCTTTACCGATGGCTCGAGGGGGCTGTTGTACACGCAAATTGTTCGCGTCCTCAAGACCA

CGCAACCCAAAGCATTCCTTCTGGAAAATGTACCAGGTCTCTTGGAAATGGGAGAAGCCCTGGAAAC
TATAGTGACGGCACTCCAAGGAGCAGGATACACCGTGACCATAGAAGTTTGTACAGCACGGTGTCTC
ACGGCACAAGCCCGCAAACGACTCTTTTTGTGGGCTTGCGCAATAACAACAACAACAACAACGACA
ATTCCGATACAGCACCGGTAGACTTTTCGTTTCCGTTTATGCCGGATTTGGGGCTACGTGCGGGAGATA
TCCTTGACTATAGCGTGGGTGATTTGGACGAAAATGGCAACCTTCCCGACAAGTACGAATTCTATCGT
ATTACCCATTCGCAAATGGACCAACTCTTGAATCGATCCAAGAGGTGGAAACCAGCAAGGCTTGCGT
GGCAAGACAAAGTCTGCGATACGATCGATGGACACTACGGTGTCACGATTGGCAAGGGAAATTCGCA
GCTTGTGCCTTCTCCGGCGCCGCTTTGTCCCCGCAGTTTTACACCTCGTGAGTGTGCGCGACTCATGGG
ATTTCCAGCTTCGTTTCGGATACCATCCGAACCAAAAGAAGGGCAGGGGAAGAGAGCCCATCTAAAG
AAACAGTACCTCATGTTTGGGAATGCCGTTTGTCCTCCGTTAATTGCGGTGTTGGCGGGTGCCGTGCTA
GCGGAGGTTTTGGGAATACGGGACTTTGCTAATCCCAACTATTGGAGAGCGAGGGGTTTGGAAGTTGG
TATTCGTATGGCATTGGATTCTTTGCTTCCCCAGCGTCAAGATGCTGTCTGCCAACGTTTGCAAAAAAT
GGGAATCATAGAATAGTGTGACAAGCACTTAGTGTGGGTTTTGCCCACAATGCTTGCTTATTGGTGTGT
CTGCGTGTCGTGCATGCTGCGGTCGGCGCAAAGACACATCAAGAAACTGGACGTCAAGTCTGGTACA
ATCCTATACATTCTTGGATGATGGGATAAAGCAAATATCGATTTTACGAGCGAATACAAACACAAAAT
CCAGAACTGAAAAAAATAAAAAGGAAACGGAAAGCCTTCTCATATTAATTTAGTATCTTCATTTTGTC
AACAA
>TRINITY_GG_2454_c3_g1_i1 len=8885 path=[0:0-3319 1:3320-3389 2:3390-8884] Gene_family="Bacterial-like DNMT"
GACATATCAGAGCAGTTGAGGCTCAACATTGCAATCGACCTAGAAAATCCCACTTTATTTTTGATTCC
AACTTGTAGCTGTTGCGACAAGTGTGGAGAACTCATTGGTTGGCGCTAGTGACCATGAAGTACGATTT
TGTCAAGGTTCAATGGGGCAGCCCATCGGAAGAACGCCTCACCAACCACTCCAAATTTGAAGCCTCA
AGTCAAGCATCCAGTTCGCTCCTAGGAAAACGGGCATCTGAGGAGAATACACGCGATGATGAAGGCG
AAGTTGAAAGTGCAGATGGTTCTAGAGAATCAACCTATAATGCTTCAAGCAACTCGTACAGTGACAC
AAATATAGCTTCACTCCTATCTTTCTCGCAGTCTGGTTCGACTCCATCGAATGAGCGGACCGAGAGTAG
ACTCCGTGATGACTCAGTCTCACCAAGGGATGATGTTTATGATGCAATTGAGACTTTCTCTGTCAGCAA
ATCTGAAAATTCTTTAGGGAACATCGCACCATCCGGGAAAACAGAAAACTCTCTCGAGTCCGTTTTGC
CTGATAAGGAAGCAGGCCATCCAGAAGGGGAGGGTGACCCCACTACGGAAAGGATGGAAAACGCAG
ACTTGAAAGTTGAGTTTGGATATCCTTCCTTTGATAAAGAAGCAGACCAGCCTACAACGGAGAAGGC
AACACAAGAAAGAGCGGAAGACGTTATCGAAGTCGAGGAAACGGTGAAAGATGAAGAAAGCATGA
AGAGTCTTCCAAACGAAGGTCCTAGAGCGGGATCTTCCAATCTCGAAGCTGACAAGCACGTCGAAGA
GAAAGCAGAAGTCACGGTAGAATCAAGTGTTCGCAGTGATAATGTATCGACTGTCGAAGAAAAGAAC
TTTAACGACAACGGAGGCATCGTTACAGATTTCTCCTTGTTGTCGCTTGAGTCACAAACGCAGAGATCT
CTCGAATTCAATACCATCGGGTCGTTCTTGTTCCAGTTATCTTTCCCTAGCAAGCAAGAAACTGAAGAA
GATGCCAAATCAGAAGGGAAAAGTTTCAACGAAGCACATTCCGCCGAAGGTGAACAAAGAACTAAT
TCGAATATAGAGGATGAAGGCCGTGAATCCCCTGAAGAAGGCCGTGAATCTCCTGATGAGGTAACGA
ACGAATTCGACAGTGGCCCTTTGCTTTCGCAAGAAGGTAGCCAACAGGCTGAAGAAAAGCTAGACGA
ATCTGCGTCGAAGCTTGACTCTGATACTATTGATTCTGATTTCCACCCTGGCAATAAAGAATTCGAAGG
CATCTTACGTGACCTTGAATCAGTTTGTGGGAAACAGCAACATAGAATTGCAATGTCTAACCCTGGAT
CTGATCACATTAATAAGCATCCTCCAAGCCAGGGACATGAGGTTCCTAATGTGACTCAGGTTTCTGAA
GAACTGAAGACCAAAGAAGACCAGATGCTAGTGGAGCAATCTGTGGTTCGTACAGAGACCGTGACTC
CCGCGCAGTCAACAAGTCAGAAGAAAAAGGGAGGACGCAGAAAATTCAGGATAAAGCGGCTCTTTT
GTGCACTAGGTCCTTGTTCCAACAAAACTGTTCTAAAGGCCGTAGAAGAAGAAAAGGATAATGAGAA
AGTTGAAAGTACCGTGAATGTTGTTGATAGCCCTGGGAAAGCTGAACTTGCTGTTGAAGAAGGATCCC
TCAAGAAAAGTTCTATGGAAGAGAGCGTGCATCAAACAAATTCGGAATCGCAAGGTGGGGTTCTATT
TAGCCCTTCAAATCAAGTCGAGCCAGTTTTAATTTGTCACTCTCTTGCATCTGCATCAAGTGCACCTCG
TCAGGCGGTTGAGCCCGCCCCTACGGAAGAACCCTCCACAGTGTCAATTGCAAGTGAACTGGGAGCA
GAAAAGGTACTGGAAGTTGACAGTAAGTATGAATCGACGAGCATTAAACAATCCTCCCAGGACGACA
TCGCCACAAGGAATGATGTCGGAGATCTTCCAGTTGCTGGGCCTTCAGAGGGGGGTTCAACAATTTCA
ACCCCAACTGAAAGTGTTCCAAGCTTCGAACCAATGAGAACTCCCGTGGTAAGGATCCTGTTGGGGG
CTTTCAAATGGCAAATGCTCAAGATCATATGGCTGCGTTTTCCCAGCAAAGTTATACGGAGAGTAACG
GGGGGAAGTATGGATCGTGGTTGTCCTCTCCAAGTGCTGCATCGCGAGGGACAGCCTCCTACACCCAA
GAAGGGACAGCTTCCTACACCCAAGAAGGGACAGCTTCCTATACCCAAGGGTCAGCTTCCTACACTC
AAGAAGGCACAGCTTCCTACACCCAAGACGGCACAGCTACTTACACCCAAGGAAGCACAATCGCATC
GGAATCGGAGGTCAGTAGCCAGTACACGTTTGATACTAGGACTGACAACGGAGAAAATCCAAGTGTT
GACCAATGCAATTCTTACATGAGTGTAAAGGAAGCAGTCAGTTACAACTTGTATGATGATGCAAGGTT
ATTTGTTAAGAGTCTAGGCTTCTCTCAAGAGGCGATTGAACAAAAGAAAGGAAACGAGCGAAAAAGC
TGGAATGCAGAATCTGCTCTGGTCAACCCTGTTTCCTCTATGTTTGGTGAGCTGGCAGACATCCTTGAG
TTTAAATTTCCTTCTACCCCAGGCAGAACATAATGGACTTTGCGATGCCTCTCGGGACATCTGGCACCA
GAAGGAAGTCATCAAGTCGCGGAGGAAACATAAGGTATCGTTAGATCTAATGACAAAGTTATTTGTA
CGTAAAGGGGTAACAGTAGGAAGTGCTCATTCAAGTGCGCCAATACGTAAAATCCACAAGAGACTCA
ATTTCCTGTCATAGCATGCCACTTTGCCGCATAGAGGCTATGTTGAGGACCTCTACTCAAAAGCTCATT
GTGTGTGCCCATTTCCACGATTATACCATCTCTTAAGACGGCCACGCGATCAGCTAGACGACCTATTGC
CCATTTGTTCGATGTTAGGAGGCTTGCTGCTCCTGTTGAACGTAATTGTTGCAATAGCTTTGATTCTTCT
ACCTCCGAAAATGAACTCGTTGCGTCGTCCAAAAGCAATAGAGAACCGAACAAAGAATGCTCCACCC
GATCGCTCGATCCAGCTGAACTATCATTATCGTACACGGCTTGAGCTATGATTTTTGATAGCAAGAGTT
TTGACCATTCGGTTGGGGACAACACCGTTGATCTTGCGTGTAGCAGAGAAGGTCGCAGTTCATCTTCA
CTGGAAGCCACGACTGTACCTAGCTTCGATGGCAGCCTAGGCAACAGCGACCACTGTAAACCGGTCA
TCTGCGAGTGTAACAAGTTCAAGGTTAACTTTAAAAAATTCGCCATTATTTCTACCGACAAACTTGCTT

ACCTGCAACGCGAGCATGATACACGACTTCTCATAAGTACCAGATGAGCTGCCACCTCTTAGAGGGTC
GATGGGCTCAAGAATTTCCTCAATTGTGGACCCAGACCACACTTTCGCAAAATCAGCAACAATTGAGA
CGTCATTTAGAAGCAAACCAAGTCTCTTCTTGAGGTGACTCCGATCCCACCTGGTGGTATCAATTCCAC
CAAGCGATATGTTTCCACGAACGAGAGTCGAGGTCAATCCTTGACTTGGTGGAAGCAGCATGGATTCC
GCTAATGCTGTCAAGAATCGTGTCTTACCAGCCCCATCATCGCCCAACACCACCAAAACTTCCCCGTT
CCGGCAGACAAAGTTAGCGCCTTTGACCGCCCATGCACGTTTGGCAGTGTAAGCTGTCCATAGATCTC
TTACCGCCAAACCTGATGTTGGATTAGCGGAAGATGGCAGGTTAGGCGTATTATTCTGTGTTCTTGACA
TTTCTTTCAATGCTAGTCGGAGTGACTGCAGAAACGCGAAAAGGCCATTTTCATTTGGCGTTGATTGAA
CCACGAAACCAAAGGTATTCCAAACCACTCGATTGGTGAAAAGCAAAGTAACACAAAGTTCCAGAA
AATACGTTTGTCGCAATCCATTCCAACTAAACGGTATTTTCAACAACAAGCCAAATGCTATAATTGGT
GCGGCAAGCATCGAAAATGACAATCCAAAATACGCCAAACGTCGTAGAAAAGTGAGCCTTGCCGAA
TTTTTCTCAAGAGGTTTAAGTAGCTTCTGGCTAAGTCGCCATCGCTCTATTGTGTCTTCGACCGCGCCGC
TTGTAGCAAGCAGAACAAGCCGACTTGCACTTGATGAGCCAAGATCAGCTACTGCTCTCGCTTTAGCC
GGTGATTCTGGATACAAACTTTCTTCATCGTTACTACTTCGTTTGGACCAAACATTTCTCCTTATTTCAA
TCATAGGAAGCACTCGAATAATGAGCAAGGCCAGCAGAATCGACAGCTGCACTCCAATCTGCAGTGG
ATGATGCCCACCTTCCACAACCTGCACCTTCACTGACGCCCAAAGATTCTTCATCGAAGAAACCAATA
TCTCCGAAATATCATTGCAGACCTTCCGAAAGTGAATTGGCCAAGAGGTGAGTGCCACCAGACGTATA
ATATCGACAGCAGATTCGTATACAGACATCAACGCCAGCCGCACTTCGGCTACACTTGTGAAAGCAA
GAGCTCCCACCACGAAGGTAACAAAAGTTTGTAGACGCGAACATTCGATCAGAGCAATAGCTTGTAA
ATGCGCAGTGTGTCCAAGAAGGGTTGGCAAGTCCGGCTCAATTCGCATTGATGATACAAGTCGCAAGT
ACAGCATACCATATTCTGCTTCGTCCTTAACAGTTGATCCAACCGAAGAAACCAGATTCCGGATTTTA
GTGCCAAATAGCAACGAGTTGGACCATGCACTAAGGAAGGCTGCCACGAAAGCAAAAGGAGCCCAA
GTGTCAAAAAAAGCAAGAAGACCTTCACGTAATGCCGTGGCATGTTCGTCCGCCCCCATTGTCGCATA
CCCTCCTAGGAGCGACGCTCTCAAACCGAGGAGAATGAATGATAAACAAACAACGTCAAGAAGTGCT
CGCGAATTTCCTATTCCTCTGCATCGTCTCGATTCCCCCGTGGTCAGCAGCAATCCATGGCCATCTTCTC
CACATCGATCGAAAAGCTTCTTCAATATATCGGGGGAACCTCCTTCTGCAGATTTCACAGAATTTCCCT
TGTATGGTGGTGGAGCAATGTACTCAGTAGGTTTGCTGCCTCGAAACAGCCTCCAAAACACCCCTGAG
CGATCTGGCTTGTCGTCGTCCGCTTCCCAAGCATCGAGGCTTGGATAGTTCTCATACGAGTAGCTTCGA
CCAATTCTCAAAACTCGCTTCAATGACGAGAAACGAGGCATAGCAGGTACAGCCTTCGCAATTCGATC
AGTCCAAGAAGTCTCTTCGTAGTCTCGACTATTCGGCAATCTACTCCTTTTCTCGTGCACATTAGCTTCT
AAGGAGCTCCTGATACCAGGTTTGCGGGGTTCGGCATTTATTGGGGGTCTGCGTCCGGCCAAGGGAGG
TCCCCTCTGCTGGTGCCTAGCTTGATTGTCTCTGCTTGGATAATGGTTGGAAGACGAGGTCCTAGATCC
TTCAGTTTTTGATGAGTCTGCTTGAGGAGTGAAGCTCATTTCGGCACTTTGCTGGCGTTGTACTGACAC
ACTTTCTGAAGTTTCAGCACTTCCAGTCCCGTAACCATTCTCTAGGCTGTTTCTTTGCTGATTACGACTG
CTGAAGCTTCCGCTAAGGCTTGGATCATGAGGCGATGAAGCACCCCCGTTGGCAGGCATTCTACTTGA
TTCCCTTGAAGGCCTGTCGCTAGGAATCACTTTTCCAAAGTTTAAGACAGCTCCACGACTAGACGTATC
GGTGCAGTGATCATCCTTCATGAATTTGTGCACAGTCGAACTGAACTCAGAAGCAGCGATTCTTTCAT
GCCTGGCATTTTCCTGCCGGGTCGACTGCTCTTTGCGATCGTTGAAACTCATGGGAGTCTTCAAGGAAT
ATTCTTGCTTTGAAAGAGGAATCTTATGGATGCCCTTGTGAGAATTCGTACTGCTGACCTTCAACAATT
GACTGATTTTCTTGATTTTCTGCTTTGGACCATTCGCACTGCCTGTCAAGAAACTTTGGTTTCTATTGGA
AACAATCGGACCTCCTTCTCCAATACCATACTTGTCCAAGATTTGTGGATCTCTATCCTTTGGAGTTTCG
TTCAGCTGTTTTGGCTGCCTTCGCAACGATGCCTGGTTATGAGTTTTGTCCTCGCCAATCGCTCGACATG
CTGGGGCTTTTGAGCTATTACCTGGTTCACCTCGTTCACCACTAATGTCACCCATAGAAAATGGTTTTTT
TCTTTCTGTCGCGACATCGTCCCCTGTTCTTCCAGCCAGCAAATCTATCGACTTTTCCACTCGAGGTGGG
GCGACTTGGATGCGCTCGCCGGTAGTTTGCTTTGATTCCCTCATATCAAGAGCATTAAAGGTTGTGGAC
GTGGCCACTTTCACGTCGCTCGGGTTCTTTGTCATATTGCCTGATCCATCAGAAGTACCGGCAGAAGTT
CGATCATCGTGGTTACTGCTAGACGATGGTAGCTTCTGAACTGAATTCACAAAAGATATTCCAGTATTG
CTCTTAATCACACCTTCCATATGCTTTGATAAGCTATCACTCGTATGTTTCATCTCCGACGGCAGGTTCG
ATTTTCCTGGTTGAATGACATGGGCCTTTGAAATGTCACGCCGTCGCAAAGGTGGCCGTGGTGGGGGT
GGCGGTTTGTTGCTCTGCTGAGCAACGCTGGCTTTTCTCCTGTCACCGTGATTGTCCACTGGCAATTTAT
CCATCTCCTTGAGGGAAATAGTCGAATGAGTCGGACCCGATTTGGAAGAAGAAACAGATTGAGATCG
CTTGCCTGTTTCAACCTCATTCACCAGATCCTCATTCATAGCCATAAAGCTATTCGTCAGTTTCTTCTCG
CCGATTTTGCCCGGAACAATGCCCTTTCCGACTGGAGGAGGTGCAAGGGAATTTCTTGGTACTTGTGC
AGTAACACCGCTGTTTGTCGATGTCATGGAGCCAAAAGTAACATCCTTTCTGATTGTAGGCGGCGCAA
TAGAGTTTCGAGGTACGCTTGCAGTAGCAGTGCTGTTTGTCAATTTCTTCTCGTTGATTGAGCCTGAAG
CAACATCCTGAGCAGGTGCAGTCACAATGCTGTTTACCAATTTCTTCTCGTTGATTGAGGCTGAAGTCA
CAGGCTTTTCGAATGGAGGAGGTGCAACAGGATTTCGTGGTACCACAACGGTGTTTGTCAATTTCGTCT
CGCTGATTGAGCCGGAAGTAGCATCCTTTCCGGTTGGAGGGGGTGCTGCAGAATTTGGAGGTGCTTCT
GCAGTCATTAAGCGGTTGGGCACTGTCCGATGATTGCGTGAGTCCGAAGTCACATCCTTTCCGATGGG
TGGAGGTGAAACAGAATTTGGCGGTGGCTCTTCAGCCACAATACGGTTGGTCAGTTTCTTCTCGTCGAT
TGAGCCCGAACTCGACTCCTTTCCTCGCAGAGGAGGTGGAACAAAAGTACGTGGTCCTTCTGCAGTGT
CATCGCTGCCTGATTTGTTCTTGCTGATTGAGCCCTTTCGGACTGGAGGAGGTGGAACAAAATCACGTG
GTACTCTTGCAGTCATACCGCTTCTTGTCAACTTCTTGTCGCTGATTGACCTCGAACTCTGATCCTTTAC
GACAGGAGGTGGTGGAACAGAAAGTCGTGGTACTTGTGCAGCCACTACGCTGTTTTTCAATTTCTTGTC
GCTGATTGAGCTTGAACTCGCATCCTTTACGACAGGAGGTGGTGGAACAGAATATCTCTTTTCTTCTGT
GGTCAAAACGCGGTTGGTCACTTTCTCTCTGCTTGAGCCCGAACGCACATCTTTTCCCATTGAAGGAAC
TGCAAGAGATTTTCTCGGCACTTCATCTTGCCTTTGATAGGACTGGTCTCTCCATCTTTGAACGCCATCG
GGAGTCTCTCCCGCAGAAGAAGAAGCTCCTTTGAGTTTCATTGTTTGCCGAGATGGTGGCATAGGACG
TATGAGTGTTGCGTTTTGCATTGTCTCAGCAAGAAGTTTGCGGCTATGGTGATGAGGTCGACCGTCTAG

AGGAGATTCATGTTTCCCACCTTTTATTTTCCTCTCATCAGCTTTGAGGTCGTTGTTTGGTAACCTCTTGG
GAGGAGGGGGAGGCACATGAGAAGACTCCTGCCTTGTCGATCTACCTGTAGCACTGGTCGTTGTAGA
ATTCCTTTCCTCTGACGGCATTGATTGTGCCTCCATCGCATCGTGAGACAACTGCGCAGTCTTCTGCTTT
CGAGCAGGTGGTGGAGGGACCTTGAAATTAGAGGGCAAATCTGGCATTGCAGGCCGTTCGACTACAG
AATCGGAGCTCTTTCGAGCTCCAGTCTTTTCGACGAGTCCACCACGGGCATCTTTTTGAGAAAAGGCC
GATTGCATGCCATCGCGGTAATCTACTGCAACATGATCCGAGCTAGCTGCAGTCACGTGCAGGGAAG
AGGACAAGAATCCTAGTGCAATCAAAACACCCAGTTGTCGGCTCATGTTAGGAAGGTGGCAGCACTA
TAGTACTCTGAAGGCGAATGTTATATAGATTGTTGTACCGACGCAGTGAAGAAACAATTTTATTATCA
CAATGCCATGTTTGGGAGTCGCAGCAAGAACGTTGTGTGATCAAACAATCAACGACTGCAAAAACGA
GATGTTTTTCGGAATCAACAACAAGACATCTCTTACATCTCTCGATCAATCAAACCAATTTCTACGGGC
ACAACCATCCCATGAGTACACTACCTGGGTAAGCGAAAAACTGACTACAAATGGCAATCCGATCAAC
ATTAGATCGTTCGTCTTACG
>TRINITY_GG_3217_c7_g1_i2 len=1132 path=[0:0-915 1:916-916 2:917-1131] Gene_family="Demethylase"
ACAACAACAACAACAACAACAACTGTACCTCATCCTTCCAAAGAGCAGCCTTTCCATGAAGACTCGG
AAACGTTCCTATCAAAGTCAGGATCAGGAGCACCAGGTCCCTTCGGCAGATGATGTGAAATCAAATA
CAATTGTCGACAAGCTACATGTCAACCAACCCTCCTCTTCTAGCGATCTCAACCCCATTCCAATCCCTA
GCGATCATCAAAGTAATAGCACCACCAAATCGACCAGTCGTTTCGTAGACGACTTGCTACAGGACCAT
GGGATTGCAGTCGAGACCATCATGACGACCAAGGGAGGTTGGTGTCTTCGAAAGGGTCTGCAGCATG
TCGTATCGGTAGAGAATGGAATCTTACGATCCTTGGTAAAACAACATGGTCCACCTCCCTTTTATCATA
CTCAACACAAATGTCGTCATGAAGCGACGGACAATCTCAAGGCACCCCAAACCTGTTTCGAGTCACTC
TGTCGGACCATAGCTGGTCAGCAATTGGCTGGCAAGGCAGCACAATCGATATGGAAAAAACTCTTGA
CAACCACAAACAACTCCTTGACTCCCAGCGTTATCATGCAATTGGCGGCTACTACGGAAAATGGCGTG
GAAATCAATTTACAAAAGCCGGCAGGATTATCACGTGCCAAGGCTCATTCGATACTCGATTTAGCCCA
ACACTTTGAACGAGGGGACTTGTCCGAAGACTTTCTCAAAACGTCATCGCAAGACAAGGTTCGAGAG
GCGCTCCTGAAAGTCAAAGGAATTGGACCATGGTCGTGTGATATGTTTGTCATGTTTTATTTGGAAGAT
CAAAATGTCTTTCCAGTGGGAGACTTGGGAGTACGAAAGGGTGTGGCTAGATTCTTTCGACTCCGAGG
GACTGGCAAAAATGCTTCTCTTTGTCCCAAAAAAAGATCTTGCCAAAATCCAGCAACTCGTCACTCCC
TTTCAGCCATATCAATCCCTCCTTACCTACTATATGTGGAGAGCCGCGGATGTCAAAGACCTGTATGTG
GATGATACTGACCCGCCCACGATGTCGACCCCATCATCCAAAAAGTCCACTTCTCCGAAGAAACAAC
GAATTGTTACCCCATAGCTAATGTAGACACATTCTATATGGTTGAG
>TRINITY_GG_1139_c1_g1_i2 len=1546 path=[0:0-1454 1:1455-1545] Gene_family="Demethylase"
TCTCCTTGGGGTTAGTGCTATCTACATTTTGTTTTCTGTTTTGTTACTGTGCAGAAGATTTTTTTCGTTTGC
GTCCTTTGGGCTGAAAGAAGGAACTTGTTTTGACTTGCTTTTCACGGTCTGCCTGCACAGCTTTCAAAA
CTTTCTTGACTCCTGATGTAACCCCCATCTCTTTCATTTGTTCCTCTGTCATCCATCGAACGTGTTTGCCA
TTGCTAGTTGTCCACGATCGTATGCTAGTAGGTTGCAAATCAATATCATGACTCGCATACTCTACCCAC
ATTGTGTGGCGCACATGACTAAAGACGTGTTCAAGGGGGGTGACCCCCTTGTTAAGACGTGTCCAAGC
AGCCTCGAAAGTGTCGCTTTCGCTGGTGCATTCTGCCAGCACATCCTCCACGAATGAGGTGAGAAGCT
TTCGACGTTTGGGGGCTGGGATTATGGGTACTTCTTTGCTGACATTCCTTTGGTTGGAGTTTTTGCTTTGA
GACGATGCAGGTTGTCGATCATCCTCGCTATTCCAAACACAAACAGACGGAAATTCCCATTGCCCTGC
CAATAAGCCTTTTTCAGGACGCTTCACTAAGAGCCATGAAGTAGTTTCTTTTGACGTATTGCTCTCCGA
AGGATTTACCCCGGACAGGACAGCAATTGCATGTACTTCTTCTCGTTTAGCAAGCTTGGGTGGAGGGA
TTGGAAAAACACTATGGCTTGCTATTTTGGCAGCCGTCTCAGGATCCATTGGAACCGCAGTATTTTCTT
TTCCGCGTTTTCCTTTGGTAGTACCTCCAATTTTTGTTTTCTCGTTCAAAAATGCATTGGAGAGATTCTCC
AAGGCTATGGATATCCCATTGTCTGCACAAAGTTTGCAAGCAGTACTACTATTAGTTGTCATGCCATCC
TGTAAAGACTCATCCAACAAACGGGTGTTGTGAGAAAAGAACGCTCCAATTTTGGTCGACAAGTAAT
AGTCTCTCAATGGATCTTGATCATCCACTCCCGTCCCCGAGGGGGAGCAATAGGTCGCTCCGAGTTCC
ATGATGGCCTGATTGACTTCTCCAGCAGCAGGGTCATTATTGTCATGTTGTGTTGTAGCGTCCATGATTT
GTTGTGCCAAATCCCAGGCTTTTCCATCTTTGACAGAGGGTGCCTTGATATGATTTGCAATCCCTCGTA
GACGACACAAGACACGACAGACATTGCCATCAACGACTGCTACTGGGACATTGTATGCAATCGAAGC
AATGGCAGCTGCTGTATAAGGACCAATTCCAGGGATGGCACGGAGTTGATCCACCGTCTGTGGCAATT
CTCCATGATGGTGGGTGACGACATACTTTGCTCCTTGATGCAACAGGCGAGCTCTCCTGTAGAAACCC
AAGCCTGCCCAATGAGAATTTACTTGTTCTTCAGTGGCATTCGCAAGATGGTGGACTGTAGGGAAGGA
TTCCATCCCTGATGGACAAGTAGGAAAACAGACAAAAAAATGAGCATGATTCTTTGGGTTCATGATG
AGATTGAAAATAACTAGAAAGTACATTATCAC
>TRINITY_GG_1141_c0_g1_i1 len=300 path=[0:0-299] Gene_family="Demethylase"
CAGATGTGGAAGTCTTATTTGTCCTTTTAGTTGTTGCCGTAGAACCATCATAAGGAGGCGGGTCACCTC
TCCAGGGAAGTTTGCGTCGATTTCTACGATACCAAGACAATAAGGCTTGGCGAATCTCGATGGCCTGC
TGTGGAGAAAAGTCATGAAAGCTTGCATCATCATGTGTCAGCCAATTTTGGACTTCATGATCGTTCTTT
GACATAGTGGCAGTAATAATAGAGATTCCTCCTCTCCTTGGAGTAAAGGTTTTTCTAGTTGCTGCCGCC
AAAAACACGTTCTGTCTACTGCTCC
>TRINITY_GG_4447_c52_g1_i1 len=1559 path=[0:0-1558] Gene_family="Demethylase"
CGACAACAAGAGAATGAATGAAAAAATTGCTGTGACGCGCGGTGGTGGAATTTTTTGGCGAAAAGAA
TGCAATATCCGAACGAACGTGGTGGAACAAGTTTTTGGACCGGAGGGTGTGTCATATGAGAAACAAG
TTGACTTCACGGTCGTCGTGGTGGTGCTATAGGACAACAAAGAGAAAACCTATTCGTACAACTACAAC
GACTAGTAGTAGTTGGTTTGTCTATTATTGTTGTTTGTTGCAGTTGAATTGTTACAATTCTTCTCCGCGTT
GTTTTTCGTCTGCTCTAGAATTATCTACTTGGGAATTGCAAAGACCAAGTTTCACCGCAGCAGCAGCAA
TGAGTAGCCGCTATCCTCTTCGAAACCGTCGTGGACTCCAGACAACATTATCGCTACAACAACATGGT
GACGAAGTTCTTTCACAATCGGACAATGTTCAAAAAGTCCATCAGGGATCAGAGGAGGTTATTCTTCC
TCCGCCTCCGAGCACTCCATCCTTGCCTACCAAACGGAGAAATGCAGTGGGACCAAAACGAAAGTCA

CCACAACAGCCGGCAGTTATCACTCCCACCTCCTCCATTGCGCCATCGTCATCATCATCATTGTCATTG
CAGAAGAACAACTACGACGACAGGAAAACCAAAGACGATGTTATTTCTCCCAAAAAACGAAAAAAG
ACAAAAGCAGTGCTACCTAATACGACTCCTCCCAAGGGATGGCAAGAAATTTATTCACTCGTGGAAG
AGCTACGGCAAGATAAGACTGCACCCGTAGATAGTGATGGAGCAGAAGCCTTACCAAACTGCAAGG
ATAATGATAGCAAAGAATATCGATTTCAGGTCCTCATTGCACTCATGTTGAGCAGTCAAACCAAAGAT
GCTGTAGTAGGTGAAGCTATGGCAAATCTACAAAACTATGGATTGAATGTTCATTCCATACAGCAAAT
TTCATCGGAAAAACTCAATCAACTCATCCGCAAGGTTGGTTTTCACAACAATAAGACAAAATACATTC
AACAAACCGTTGAAATATTACTTTCGGACCATGATGGGGATATTCCCAAGACGGCCAAGGAAATGAT
GGAATTGCCAGGAATTGGTCCTAAAATGGCCTTTTTAATTGAGACCATTGCTTGGGGGACCACTTCTGG
CATTGGAATTGATACTCATATGCATCGAATGTTTAATCAATTGAAATGGGTCCAAAGCAAATCACCCG
AACAAACGCGACTTCAATTGCAAGCCTGGTTTCCAAGAGAACGTTGGTCGACTGTCAATTATTTATGG
GTTGGTTTTGGACAAGAAGTCCAACAACAAAAACCGAAAATGCTGCAAAAGGTTTTATCTTGTAGTCG
ACCAAAAGAGGCCTTGAATCTAGTGAAACGCCTGGGATTAGATTACAAAAAGGAAGGAGCCAAGCTT
GGTCTTGAAGAAGCCATTCGACAAGCCCTCTTGAAAGATACAAAGGATCGTCTCCACACGGATATAC
AAATCAGCGACAGCCAAATGTAACTTTTCAGATATACCGTAGTCTGACGCACATACATTGTAACTCAT
